# Nutrition, Exercise, and Wellness Treatment in bipolar disorder: proof of concept for a consolidated intervention

**DOI:** 10.1186/2194-7511-1-24

**Published:** 2013-10-25

**Authors:** Louisa G Sylvia, Stephanie Salcedo, Emily E Bernstein, Ji Hyun Baek, Andrew A Nierenberg, Thilo Deckersbach

**Affiliations:** Department of Psychiatry, Massachusetts General Hospital, 50 Staniford St, Suite 580, Boston, MA 02114 USA; Harvard Medical School, Boston, MA 02115 USA

**Keywords:** Bipolar disorder, Exercise, Behavior therapy, Cognitive behavior therapy, Cardiovascular disease

## Abstract

**Background:**

This pilot study examines the proof of concept of a consolidated Nutrition, Exercise, and Wellness Treatment (NEW Tx) for overweight individuals with bipolar disorder.

**Findings:**

Five participants completed NEW Tx, a 20-week individual cognitive behavioral therapy-based treatment comprising three modules: Nutrition teaches appropriate serving sizes and balanced diet; Exercise emphasizes increasing weekly physical activity; Wellness focuses on skills for healthy decision-making. Participants attended most sessions and reported high satisfaction with the treatment. Participants' weight, cholesterol and trigyclerides decreased over the study duration as well as number of daily calories and sugar intake. We found that weekly exercise duration more than tripled over the study duration and depressive symptoms and functioning have improved.

**Conclusions:**

These results offer proof of concept that consolidated NEW Tx is feasible and acceptable and has the potential to improve nutrition, exercise, wellness, and mood symptoms in bipolar disorder. Future iterations of NEW Tx will reflect the strengths and lessons learned from this study.

## Findings

Individuals with bipolar disorder experience a disproportionately high incidence of metabolic syndrome and other cardiovascular risk factors such as obesity, diabetes, hypertension, and dyslipidemia, in addition to the chronic cognitive and emotional burden of manic, hypomanic, and depressive symptoms (McIntyre et al. 2005; Kilbourne et al. 2004; Soreca et al. 2008). Physical inactivity and poor eating habits are common in this population and compound the effects of poor physical and mental health (Fagiolini et al. 2008). Medical comorbidities, obesity, and inactivity are associated with depression, worse course of illness, treatment noncompliance, worse treatment outcomes, and greater suicidality for patients with bipolar disorder (Fagiolini et al. 2003).

Although pharmacotherapy remains the principal treatment for bipolar disorder, side effects can frequently increase the risk of cardiovascular disease (Serretti et al. 2013; De Almeida et al. 2011; Ketter 2010). There is a need for adjunctive therapies to lessen residual mood symptoms and the debilitating medical burden associated with the illness. For example, there is growing evidence to support the efficacy of exercise as an adjunct treatment for bipolar disorder. Ng et al. (2007) found that bipolar inpatients who participated in a walking group 5 days per week for 40 min per session reported lower depression and anxiety symptoms than those who did not. Additionally, an acute bout of exercise (i.e., walking on a treadmill for 20 min at 70% of an individual’s maximum heart rate) significantly improved bipolar participants’ mood (Hays 2008).

Obesity, a risk factor for cardiovascular and endocrine diseases, is a major source of the medical burden associated with bipolar disorder (Fagiolini et al. 2008). Yet, pharmacological strategies (i.e., sibutramine, topiramate) as adjunctive treatments for psychotropic-associated weight gain have been examined in overweight or obese outpatients with bipolar disorder with limited benefit, perhaps due to low adherence (<21%) (McElroy et al. 2007). Even less promising data has been observed in pediatric bipolar samples (Correll 2007) and schizophrenia (Faulkner et al. 2007). Thus, adjunct psychosocial interventions have become increasingly important in reversing the behavioral antecedents that lead to poor nutrition and overeating. Psychosocial interventions, including interpersonal therapy (Tanofsky-Kraff et al. 2007), cognitive behavioral therapy (Devlin et al. 2005; Fairburn et al. 1993), and behavioral modification (Brownell 2000), have been developed and are effective in reducing weight in healthy populations. The evidence for these interventions for individuals with severe mental illness is promising, but psychosocial interventions continue to be vastly underdeveloped and understudied given this population’s increased risk for developing metabolic syndrome (Ganguli 2007).

This is particularly problematic, as reducing the medical burden in bipolar disorder requires a specialized approach given their unique needs (lack of motivation, higher rates of substance use, less stable incomes, cognitive impairment) (Stauffer et al. 2009; Velligan et al. 2000; Casagrande et al. 2010). Moreover, many interventions have substantial limitations (Miller 1999). For example, the Lifestyle Challenge Program for bipolar disorder incorporated nutrition, weight-management, and exercise strategies into weekly group-based psychoeducational sessions (Malone et al. 2005), but only 43% of the participants completed the program and risk factors for cardiovascular disease were not examined (Malone et al. 2005). Similarly, the Integrated Intervention Model, an individualized psychoeducational program on sleep/wake rhythms, nutrition, exercise, and time management, improved overall functioning and reduced psychiatric hospitalizations, but also did not examine risk factors for cardiovascular disease (Fagiolini et al. 2008). A pilot study of the In SHAPE program, an individualized health promotion program for individuals with serious mental illness, found that participation resulted in increased physical activity, reduction in waist circumference, and improvement in mental health functioning, but there were no objective daily measures of participants’ dietary intake (i.e., food diaries) (Van Citters et al. 2010).

Most recently, Daumit et al. (2013) found that their lifestyle intervention for serious mental illness significantly reduced weight by 1.7% at 6 months and 3.4% at 18 months. Although the treatment group consistently lost weight over the study duration, further research building on these promising data is warranted to yield more robust changes in weight. Other current treatments for co-morbid medical and psychiatric conditions focus on establishing co-located general medical providers or treatment teams within mental health clinics (Lehman et al. 2004; Druss et al. 2001). Although these approaches are targeting medical burden and enhance communication between providers, they tend to be costly, rely on an abundance of resources, or do not develop an individualized, integrated lifestyle program for SMI (Miller 1999).

Nutrition, Exercise, and Wellness Treatment (NEW Tx) is a proposed solution to this problem as it builds upon previous empirical studies in healthy populations (Svetkey et al. 2003; Brownell 2000; Bray et al. 1999), as well as the limited literature on lifestyle modification with bipolar populations. For example, specific reward-response strategies from the Diabetes and Prevention Program (DPP) were included in NEW Tx to increase adherence to the manual or motivation. Other examples of proven strategies from the DPP that were included in NEW Tx are the following: (1) session structure (e.g., reviewing homework from the previous week, discussing educational material, doing an activity, setting a goal for the next week, assigning homework); (2) inclusion of 'affirmations’ or positive self-talk; (3) self-monitoring; (4) the 'plate method’ to teach basic food nutrition or a balanced diet; (5) 'navigating how to eat out’ or tips for eating healthy when not eating at home or making their own meals; and (6) involving family and friends in the weight loss programs (Bray et al. 1999). In short, NEW Tx is a lifestyle change program that integrates proven intervention components from the general population to target individuals with bipolar disorder to provide clinically meaningful changes in patient-centered outcomes.

The goal of this study is to test whether a novel consolidated treatment is feasible, well tolerated, and can result in sustainable weight loss, improved exercise and eating habits, reduced medical comorbidity, and potentially improve the course of illness of bipolar disorder.

### Methods

#### Participants

Adult participants were recruited from the Massachusetts General Hospital. Eligible participants had a primary diagnosis of bipolar disorder, as determined by the clinician-administered Mini International Neuropsychiatric Interview (MINI Plus) (Sheehan et al. 1998). Participants were symptomatic (Clinical Global Impression-Bipolar, CGI-BP ≥ 3) and overweight or obese (BMI ≥ 25). To ensure patient safety, all potential participants completed the Physical Activity Readiness Questionnaire (PAR-Q) (Thomas et al. 1992) which assesses any risk involved in their starting an exercise program; if an individual endorsed any contraindication to physical activity, approval from his or her physician was required before enrolling. Five participants (3 females, 1 Hispanic/Latino) ages 23 to 64 years (*M* = 44, SD = 16) completed the study between 2012 and 2013. Four participants were diagnosed with bipolar I disorder. One participant reported having an income of US$24,999 or less, two reported earning between US$25,000 and US$49,999, one reported earning between US$50,000 and US$74,999, and one earned over US$75,000. The study protocol was approved by the Partners Human Research Committee, and participants provided informed consent prior to initiation of any study procedure.

#### Summary of intervention

This is an 18-session, 20-week cognitive behavioral therapy (CBT)-based treatment comprising three modules: Nutrition, Exercise, and Wellness (NEW Tx). The first module, Nutrition, aims to maximize weight loss and improve nutritious eating. Thus, we discuss portion sizes, anti-craving strategies, the concept of a 'balanced diet’, and maintaining such a diet, as well as essential vitamins and minerals, particularly ones that may be beneficial for mood disorders. The goal of the second module, Exercise, is to reach a healthy level of weekly exercise or exercise of moderate intensity, at 5 days per week, for 30 min each day (Heath 2005). This module begins by discussing the importance and rationale for exercise, particularly its ability to enhance mood for individuals with bipolar disorder. We also discuss ways to increase *lifestyle activity* (e.g., taking the stairs, standing instead of sitting, walking to the store), as opposed to 'exercising’, to stress that exercise is a task not requiring sustained or extreme energy, concentration, and motivation, but it can be a more casual experience that can last for a relatively short period of time and still be helpful. The last module, Wellness, is composed of CBT-based sessions (i.e., cognitive restructuring, problem solving strategies) that focus on reinforcing the importance of making healthy decisions (i.e., food choices, exercise, reduction of substance use/caffeine, smoking, sleep) and increasing adherence to the nutrition and exercise modules. The detailed rationale and description of the intervention are described elsewhere (Sylvia et al. 2011).

#### Procedures and analyses

Expectations and satisfaction were measured at baseline and study exit with the NEW Tx Scale, a novel five-point Likert Scale self-report measure consisting of ten items (e.g., 'This treatment will be helpful/was helpful for losing weight’, 'I will learn/learned skills to change my unhealthy habits’). The Client-Satisfaction Questionnaire (CSQ-8) (Nguyen et al. 1983) assessed participant acceptability with the treatment at study exit. At each visit, participants reported their exercise activity (frequency, duration, and type) from the prior week and returned their daily food diaries.

At weeks 0 and 20, depressive and manic/hypomanic symptoms, as well as overall illness severity, were assessed using the Montgomery Asberg Depression Rating Scale (MADRS), Young Mania Rating Scale (YMRS), and CGI-BP (Spearing et al. 1997). The Range of Impaired Functioning Tool (LIFE-RIFT; Leon et al. 2000), administered at weeks 0 and 20, assessed the extent to which the medical burden has impacted psychosocial functioning in various domains, including work, school, interpersonal relationships, recreational activities, and overall life satisfaction. The Clinical Monitoring Form (CMF) Medication module recorded participants’ weekly medication usage and dosage changes (Sachs et al. 2002). Lab results and vital signs data were also collected at pre-and post-treatment.

A pilot study is not a hypothesis-testing study, but a necessary initial step in exploring a novel intervention to inform its feasibility and identify modifications needed in the design of a larger ensuing hypothesis-testing study. Thus, it would be premature to conduct inferential statistics at this time and instead, for each measure, we calculated the mean, standard deviation, 95% confidence interval (CI), and then used Cohen’s *d* effect sizes for repeated measures to determine the magnitude of the changes we observed (Leon et al. 2011).

### Results

#### Feasibility and acceptability

Participants attended 85% of the 18 NEW Tx sessions (*M* = 15.4, SD = 1.95, range = 12 to 18) and completed food diaries in 80% of the time nearly every week (*M* = 14.4, SD = 4.54, range = 7 to 19). Of note, one participant was an outlier (i.e., completed only 12 sessions and seven weekly food diaries) due to spending several weeks out of the country for a family emergency. Participants reported high expectations for the intervention (pre-treatment NEW Tx Scale *M* = 46.4 out of 50, SD = 4.16) and high satisfaction with the treatment (post-treatment NEW Tx Scale *M* = 45.5 out of 50, SD = 2.08, CSQ-8 *M* = 30.4, SD = 2.07).

#### Preliminary effectiveness

At study entry, four participants were taking mood stabilizers, and three were on anxiolytics/hypnotics, antidepressants, and antipsychotics. One participant did not have medication information available at study entry, but reported taking a mood stabilizer, anxiolytic, antipsychotic, and antidepressant at week 10 (mid-treatment). Outcome variables are presented in Table 1. Participants increased their daily servings of vegetables and consumed fewer daily servings of sweets (e.g., jam, syrup, candy), calories, and sugars over the study duration. Participants’ weight, cholesterol, high-density lipoprotein (HDL) cholesterol, low-density lipoprotein (LDL) cholesterol, triglycerides, and plasma glucose decreased over the study duration. Participants’ exercise more than tripled over the study duration and depressive symptoms and overall functioning also improved (Table 1).

**Table 1:** **Changes in nutrition, exercise, medical comorbidities, and mood symptoms**

	Variable	Baseline***M***(SD)	Week 20***M***(SD)	***d***value	95% CI
Nutrition	Number of vegetable servings (per day)	2.2 (1.2)	2.5 (1.4)	0.86	-0.8 to 0.1
	Number of sweets servings (per day)	1.4 (1.6)	1.2 (0.8)	0.26	-0.9 to 1.3
	Calories (per day)	1,847.0 (613.0)	1,598.0 (387.5)	0.28	-838.7 to 1,336.7
	Sugars (g/day)	108.3 (55.0)	75.1 (37.1)	0.67	-28.0 to 94.4
Exercise	Exercise frequency (days/week)	1.6 (1.5)	4.1 (1.8)	1.74	-6.5 to 1.5
	Exercise duration (min/week)	75.0 (77.9)	264.2 (60.8)	2.00	-452.2 to 73.9
Medical comorbidities	Weight (lbs)	171.4 (35.4)	169.7 (36.9)	0.59	-1.9 to 5.3
	Body mass index (kg/m^2^)	28.4 (5.6)	28.3 (5.2)	0.03	-0.9 to 0.9
	Waist circumference (cm)	39.5 (4.3)	38.0 (5.0)	0.48	-2.4 to 5.4
	Cholesterol (mg/dL)	197.2 (35.6)	179.6 (50.9)	0.63	-149.3 to 258.9
	HDL cholesterol (mg/dL)	54.4 (14.1)	49.2 (13.0)	0.69	-4.2 to 14.6
	LDL cholesterol (mg/dL)	115.4 (41.3)	88.0 (34.9)	0.72	-19.6 to 74.4
	Triglycerides (mg/dL)	158.2 (87.0)	108.0 (20.3)	0.67	-43.0 to 143.4
	Plasma glucose (mg/dL)	87.8 (10.3)	83.2 (7.5)	0.91	-1.6 to 10.8
Mood symptoms and functioning	MADRS	17.2 (5.2)	13.2 (10.1)	0.26	-15.7 to 21.7
	YMRS	4.4 (2.0)	5.6 (3.9)	0.59	-3.3 to 1.7
	CGI-Mania	1.4 (0.9)	2.0 (0.7)	0.53	-2.0 to 0.8
	CGI-Depression	3.6 (0.6)	2.8 (1.3)	0.62	-0.8 to 2.4
	CGI-Overall	3.8 (0.5)	3.2 (0.8)	0.67	-0.5 to 1.7
	LIFE-RIFT	12.0 (3.1)	9.4 (2.1)	1.26	0.0 to 5.2

MADRS, Montgomery Asberg Depression Rating Scale; YMRS, Young Mania Rating Scale; CGI-Mania, Clinical Global Impression-Mania subscale; CGI-Depression, Clinical Global Impression-Depression subscale; CGI-Overall, Clinical Global Impression-Overall Bipolar Illness; LIFE-RIFT, Longitudinal Interval Follow-up Evaluation-Range Impaired Functioning Tool.

### Discussion

High attendance and satisfaction ratings suggest that NEW Tx was feasible and acceptable. Participants entered the study with poor exercise and nutritional habits and ate fewer than the recommended daily servings of fruits and vegetables, as indicated by the US Department of Health’s dietary guidelines (Ahmed and Blumberg 2009); however, participants showed improvement in both domains. The changes in participants’ vegetable and sugar consumption resulted in medium to large effect sizes, but only changes in sugar consumption appear to be clinically meaningful (i.e., a reduction of 33.2 g/day). In contrast, changes in calories yielded a small effect, but was a clinically meaningful change (or nearly <250 cal/day). In regards to changes in exercise, it is not surprising that we saw very large effect sizes, as this has consistently been one of the most robust and clinically meaningful changes associated with NEW Tx (Sylvia et al. 2011).

Participants also demonstrated improvements in their medical comorbidities, depressive symptoms, and overall functioning. Unfortunately, we did not see clinically meaningful improvement in body weight over the study duration or losing at least 5% of one’s baseline weight; however, improvements in cholesterol, triglycerides, and glucose yielded medium to large effect sizes and corresponded with robust changes. It is likely that we did not see the expected weight loss, as the average body mass index at study entry was only 28.4, which is just slightly overweight, and therefore does not allow much room for improvement on this metric over the study duration. Interestingly, a four-point change in the MADRS depression score from pre-to post-treatment corresponded with a small-medium effect size but seems clinically meaningful given that a six-point difference in MADRS scores yields a difference in the interpretation of one’s severity of depression (i.e., 0 to 6, normal/symptoms absent; 7 to 19, mild depression; 20 to 34, moderate depression; >34, severe depression). Changes in depression are also highlighted by nearly a one-point improvement on the CGI-Depression Scale corresponding with a medium effect and robust change. Participants’ nearly three-point improvement in LIFE-RIFT scores corresponds to a large effect size as well as a clinically meaningful change. The results of this study also mimic our previous trial with NEW Tx (Sylvia et al. 2011) in that the level of functioning (i.e., LIFE-RIFT) improved from mild/moderate impairment to none or 'in recovery’ (mean post-treatment LIFE-RIFT score = 9) (Leon et al. 2000). Of note, YMRS and CGI-Mania scores increased over the study duration. These data support other recent findings that more frequent exercise is associated with greater manic symptom severity (Sylvia et al. 2013); however, it is also possible that this is a chance finding as these changes were not clinically meaningful. In short, further research is warranted to investigate the association of elevated mood and exercise/lifestyle interventions.

Results should be considered in the context of a few key limitations. First, this was an open trial with no control group or blinded raters. Second, the small sample size limits our ability to draw stronger conclusions about efficacy. Third, participants chose to participate in a health and wellness study, suggesting that they could be more motivated to make such changes than the general bipolar population. This study also did not include a follow-up visit, limiting our ability to measure potential relapse amongst participants. Nonetheless, this study provides proof of the concept that NEW Tx has the potential to help patients with bipolar disorder make lifestyle changes to improve their physical health as well as yield positive outcomes for their mental health.

### Conclusions

Further research is warranted to investigate the effectiveness of NEW Tx; however, the present study yields preliminary information on the dietary composition of individuals with bipolar disorder as well as highlights the promise of NEW Tx. Strengths of NEW Tx include a flexible, three-module format, detailed food diary analysis, and CBT skills tailored to bipolar disorder (e.g., cognitive restructuring specific to eating and exercising, discussion of medication side effects, creating polar-specific goals and weekly schedules, problem solving obstacles of adherence) to assist with making healthy lifestyle changes. Incorporating modules focused on both nutrition and physical activity addresses key habits to prevent cardiovascular disease while integrating the Wellness module (e.g., CBT skills to maximize adherence and assist with making other healthy lifestyle changes) throughout the treatment reinforces skills for making healthier daily choices. Detailed weekly food diary analysis allowed the clinician to provide specific, concrete feedback to participants regarding progress and areas for improvement in their diet.

To maximize the potential effectiveness of this intervention, we will assess a revised version of NEW Tx in a randomized, controlled trial and reflect lessons learned from this pilot study, for example, including more motivational interviewing techniques (Miller and Rollnick 2002) to improve intrinsic motivation of participants in NEW Tx, as well as other response-reward strategies (e.g., the use of 'celebrations’ or rewards for participants’ accomplishments as this seemed effective in the DPP lifestyle intervention) (Bray et al. 1999). Persistent sleep problems may have also interfered with functioning, nutrition, and exercise, indicating a need for more structured sleep hygiene in the Wellness module. We also expect to further improve adherence by working with participants to identify social supports and incorporating more motivational interviewing into the manual. In summary, these data suggest that NEW Tx is both feasible and acceptable and has the potential to reduce the medical burden associated with bipolar disorder.
